# Maternal Exposure to PM_2.5_ during Pregnancy Induces Impaired Development of Cerebral Cortex in Mice Offspring

**DOI:** 10.3390/ijms19010257

**Published:** 2018-01-16

**Authors:** Tianliang Zhang, Xinrui Zheng, Xia Wang, Hui Zhao, Tingting Wang, Hongxia Zhang, Wanwei Li, Hua Shen, Li Yu

**Affiliations:** 1 Experimental Center for Medical Research, Weifang Medical University, Weifang 261053, China; zhangtl@wfmc.edu.cn; 2 School of Clinical Medicine, Weifang Medical University, Weifang 261053, China; zhengxr@wfmc.edu.cn (X.Z.); zhaoh@wfmc.edu.cn (H.Z.); wangtt@wfmc.edu.cn (T.W.); zhanghx@wfmc.edu.cn (H.Z.); 3 School of Public Health and Management, Weifang Medical University, Weifang 261053, China; wangxia@wfmc.edu.cn (X.W.); liwanwei80@wfmc.edu.cn (W.L.); 4 Department of Mathematics and Statistics, University of Calgary, Calgary, AB T2N 1N4, Canada; hua.shen@ucalgary.ca

**Keywords:** PM_2.5_, offspring, cerebral cortex, caspase-3

## Abstract

Air pollution is a serious environmental health problem closely related to the occurrence of central nervous system diseases. Exposure to particulate matter with an aerodynamic diameter less than or equal to 2.5 µm (PM_2.5_) during pregnancy may affect the growth and development of infants. The present study was to investigate the effects of maternal exposure to PM_2.5_ during pregnancy on brain development in mice offspring. Pregnant mice were randomly divided into experimental groups of low-, medium-, or high-dosages of PM_2.5_, a mock-treated group which was treated with the same amount of phosphate buffer solution (PBS), and acontrol group which was untreated. The ethology of offspring mice on postnatal days 1, 7, 14, 21, and 30, along with neuronal development and apoptosis in the cerebral cortex were investigated. Compared with the control, neuronal mitochondrial cristae fracture, changed autophagy characteristics, significantly increased terminal deoxynucleotidyl transferase dUTP nick end labeling (TUNEL) positive cell rate, and mRNA levels of apoptosis-related caspase-8 and caspase-9 were found in cerebral cortex of mice offspring from the treatment groups, with mRNA levels of Bcl-2 and ratio of Bcl-2 to Bax decreased. Treatment groups also demonstrated enhanced protein expressions of apoptosis-related cleaved caspase-3, cleaved caspase-8 and cleaved caspase-9, along with declined proliferating cell nuclear antigen (PCNA), Bcl-2, and ratio of Bcl-2 to Bax. Open field experiments and tail suspension experiments showed that exposure to high dosage of PM_2.5_ resulted in decreased spontaneous activities but increased static accumulation time in mice offspring, indicating anxiety, depression, and social behavioral changes. Our results suggested that maternal exposure to PM_2.5_ during pregnancy might interfere with cerebral cortex development in mice offspring by affecting cell apoptosis.

## 1. Introduction

The negative impact of environmental pollution on health has been drawing more and more attention in recent decades. According to the American Environmental Protection Administration (EPA) (2015), the major air pollutants include NO_x_, SO_2_, O_3_, PM_2.5_, and PM_10_. PM_2.5_ refers to fine particles with an aerodynamic diameter less than or equal to 2.5 μm [1]. It consists of complex constituents including heavy metals and toxic organic pollutants, and there are several investigations concerning the effects of exposure to air pollutants on admission rates, mortality rates and prognosis of respiratory diseases, cardiovascular disease, and stroke [2,3,4,5,6]. Air pollution has become an independent risk factor for cardiovascular diseases and numerous studies have reported the correlations between atmospheric particulate matter exposure and oxidative stress, lung and systemic inflammation, endothelial dysfunction, atherosclerosis, and cardiac autonomic dysfunction [7,8,9].

Epidemiological studies suggest that air pollution affects cognitive function [10] and is also related to central nervous system diseases such as Alzheimer’s disease, Parkinson’s disease, amyotrophic lateral sclerosis, multiple sclerosis, stroke, and so on [11,12,13]. More and more studies have focused on the developmental toxicity of air pollutants. It refers to the deleterious effects (such as structural abnormalities, growth retardation, dysfunction, and death) exerted on prenatal offspring, resulting from maternal and/or maternal contact with exogenous physical and chemical factors. The tiny particulate matters which can go directly into the alveoli of lung could penetrate the blood-gas and placental barriers [14], thus are potentially dangerous to a fetus. Epidemiological studies showed that exposure to air pollutants during pregnancy could lead to preterm and increased mortality of preterm infants [15]. Air pollution appeared to exert adverse effects on brain maturation during a critical period, with changes in specific functional domain [16]. 

Exposure to non-genetic factors, such as environmental ones, has been proven to interfere with nervous system development. It was shown that prenatal and neonatal exposure to traffic- related air pollutants could result in adult behavioral and cognitive impairments [17]. Clinical cohort and animal studies suggested that prenatal exposure to particulate air pollutants led to increased risks of brain development disorders such as autism spectrum disorders and schizophrenia in offspring [18,19,20].

To the best of our knowledge, there are few studies concerning the effects of maternal exposure to PM_2.5_ during pregnancy on the development of the cerebral cortex in mice offspring. The present study was to establish a PM_2.5_ trachea drip animal model for pregnant mice and investigate the effects of exposure to PM_2.5_ during pregnancy on the apoptosis-related genes and their expressions in cerebral cortex neurons in mice offspring, and then the potential mechanism by which exposure to PM_2.5_ during pregnancy impairs the development of cerebral cortical. 

## 2. Results

### 2.1. Exposure to PM_2.5_ during Pregnancy Caused Pathological Changes of Cerebral Cortex in Mice Offspring

**Figure 1:**
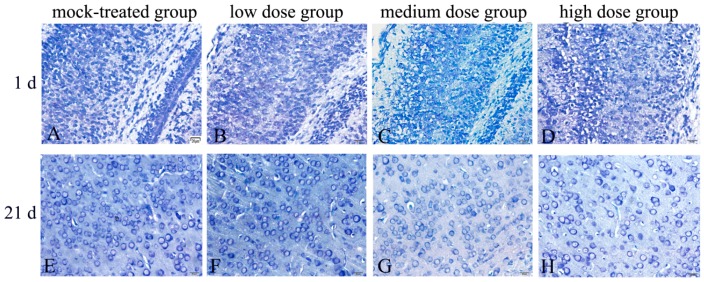
Morphological changes of cerebral cortex in mice offspring after maternal exposure to PM_2.5_ during pregnancy (Nissl staining). (**A**–**D**) mice offspring on postnatal day 1 after birth; (**E**–**H**) mice offspring on postnatal day 21 after birth. (**A**,**E**) mock-treated group; (**B**,**F**) low-dosage group; (**C**,**G**) medium-dosage group; (**D**,**H**) high-dosage group. Bar = 20 μm.

The results of Nissl’s staining (Figure 1) demonstrated that there were large number of neurons in the cerebral cortex of mice offspring on postnatal days 1, 7, 14, 21, and 30 in mock-treated group. The neurons were structurally integrated with large cell bodies, regular arrangement and compact connection. Neuron Nissl bodies were deep and cytoplasm was abundant. With the increase of PM_2.5_ exposure dosage, gradually reduced neurons, disordered arrangements, smaller cell bodies, lightly stained Nissl bodies, and decreased cytoplasm were observed, compared with the control group (Table 1). Therefore, evident pathological changes occurred in brain tissues of mice offspring exposed to PM_2.5_ and the damages became much more serious with exposure dosage. 

### 2.2. Ultrastructural Changes of Cerebral Cortical Neurons in Newborn Mice Offspring

**Figure 2:**
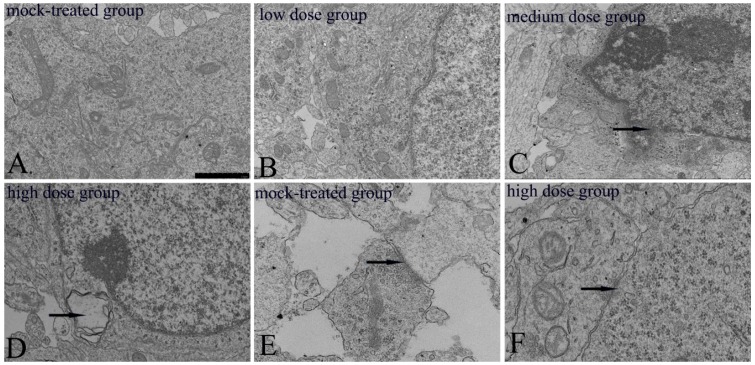
Ultrastructural changes of cerebral cortex neurons and synapses in mice offspring after maternal exposure to PM_2.5_ during pregnancy. (**A**) mock-treated group, normal neuron; (**B**) low-dosage group, no significant changes in the neuron; (**C**) The arrow shows the indistinct nuclear membrane; (**D**) the arrow shows the autophagic body; (**E**,**F**) the arrow shows the synapse. Bar = 1.0 μm.

Ultrastructures of mitochondria in cerebral cortex neurons of mice offspring from both the mock-treated group and the high-dosage group on postnatal day 1 were observed to study the potential impairment of exposure to PM_2.5_ on mitochondria which plays a vital role in apoptosis. It could be learned that neurons in the mock-treated group demonstrated abundant mitochondria with intact capsule, regular, continuous, and dense cristae arrangement (Figure 2A). And no obvious ultrastructural changes of neurons in the low-dosage group were found (Figure 2B). Compared with the mock-treated group, obvious ultrastructural changes, including broken and partly blurred mitochondrial cristae, fuzzy and broken nuclear membrane, and autophagic bodies in cytoplasm, occurred in the cerebral cortex neurons of the medium and high-dosage groups (Figure 2C,D), indicating certain effects of exposure to high dosage of PM_2.5_ during pregnancy on mitochondrial function of neurons in mice offspring. As a key part of message transmission, synapse in the PM_2.5_ high-dosage group presented a decreased number of synaptic vesicles and presynaptic and postsynaptic densities of membranes (Figure 2F), compared with the mock-treated group (Figure 2E). The number of presynaptic vesicles were 18.5 ± 2.64 (mock-treated group) and 10.8 ± 2.39 (high-dosage group), with statistically significant difference at *p* < 0.01. Ultrastructural changes further suggested that exposure to PM_2.5_ during pregnancy could exert obvious impairments on brain tissue in mice offspring. 

### 2.3. Terminal Deoxynucleotidyl Transferase dUTP Nick End Labeling (TUNEL)

**Figure 3:**
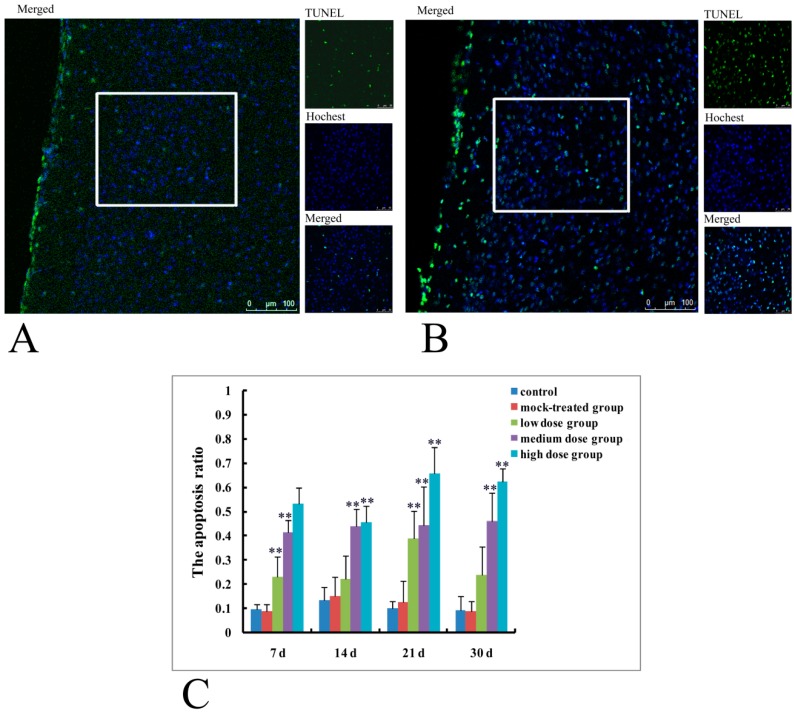
Apoptosis of cerebral cortex neurons in mice offspring after maternal exposure to PM_2.5_ during pregnancy. (**A**) TUNEL of mice offspring on postnatal day 14 from mock-treated group; TUNEL, Hochest and merged figures were derived from the area marked by the white box; (**B**) TUNEL of mice offspring on postnatal day 14 from high-dosage group; TUNEL, Hochest and merged figures were derived from the area marked by the white box; (**C**) the apoptotic ratio of nerve cells. Bar = 20 μm. ** *p* < 0.01, compared with control.

Results of TUNEL were shown in Figure 3 from which it could be seen that TUNEL positive cells in the cerebral cortex of mice offspring on postnatal day 14 increased significantly in high-dosage group (Figure 3B), compared with fewer ones in mock-treated group (Figure 3A). Apoptosis ratios in cerebral cortex of mice offspring on postnatal days 7, 14, 21, and 30 from different dosage groups were shown in Figure 3C.

TUNEL positive rates for mice offspring on postnatal days 7 and 21 from the low-dose group were significantly different from that of the control (*p* < 0.01). TUNEL positive rates of the medium and high- dosage groups at all of the four time points (postnatal days 7, 14, 21, and 30) were significantly different from those of the control (*p* < 0.01). These results indicated that exposure to PM_2.5_ (medium and high-dosage especially) during pregnancy resulted in significantly increased apoptosis rates in cerebral cortex of mice offspring.

### 2.4. Exposure to PM_2.5_ during Pregnancy Caused Elevated Expressions of Apoptosis-Related Genes in Cerebral Cortex of Mice Offspring

#### 2.4.1. Effects of Exposure to PM_2.5_ on mRNA Levels of Caspase-3, -8, and -9 and Protein Expressions of Caspase-3, Cleaved Caspase-3, -8, and -9

**Figure 4:**
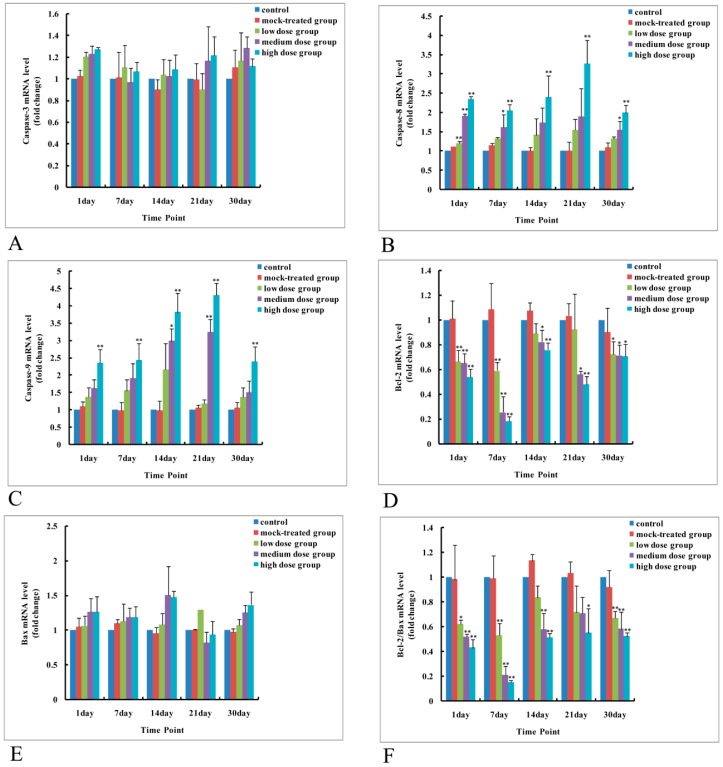
mRNA level changes of apoptosis-related genes in cerebral cortex of mice offspring after maternal exposure to PM_2.5_ during pregnancy. (**A**) Caspase-3; (**B**) Caspase-8; (**C**) Caspase-9; (**D**) Bcl-2; (**E**) Bax; (**F**) ratio of Bcl-2 to Bax. * *p* < 0.05, compared with control; ** *p* < 0.01, compared with control.

**Figure 5:**
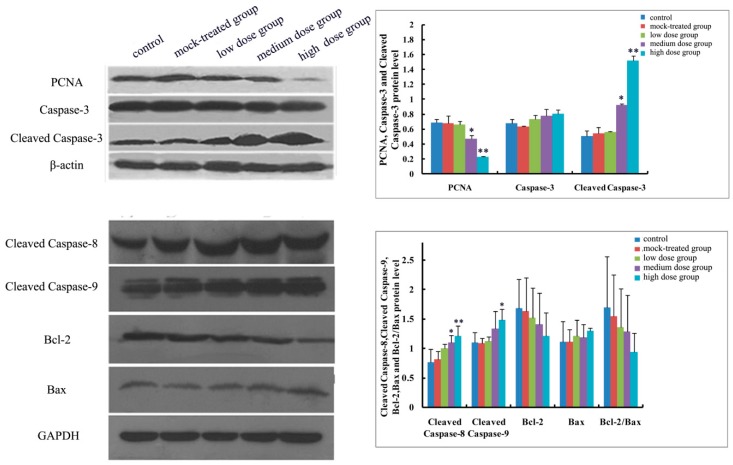
Expressions of apoptosis-related proteins (Caspase-3, Cleaved Caspase-3, Cleaved Caspase-8, Cleaved Caspase-9, Bcl-2, Bax, and Bcl-2/Bax) and proliferating cell nuclear antigen (PCNA) in cerebral cortex of mice offspring on postnatal day 1 after maternal exposure to PM_2.5_ during pregnancy. Left: the results of Western blot; Right: histogram of relative protein levels. * *p* < 0.05, compared with control; ** *p* < 0.01, compared with control.

**Figure 6:**
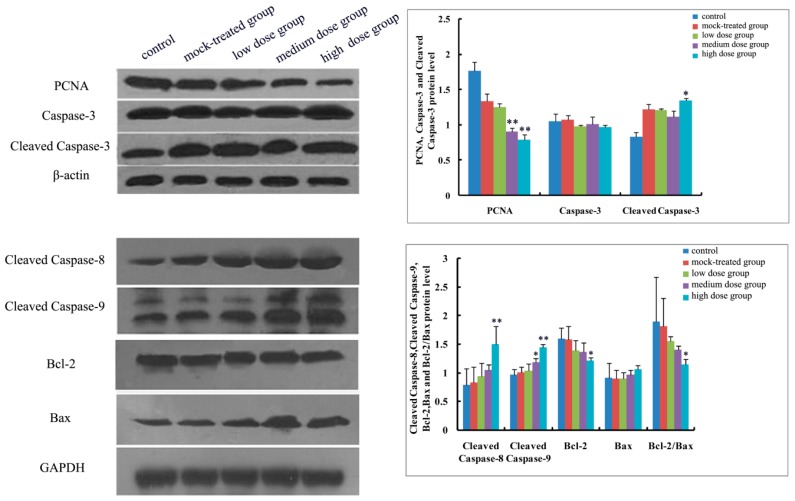
Expressions of apoptosis-related proteins (Caspase-3, Cleaved Caspase-3, Cleaved Caspase-8, Cleaved Caspase-9, Bcl-2, Bax, and Bcl-2/Bax) and PCNA in cerebral cortex of mice offspring on postnatal day 7 after maternal exposure to PM_2.5_ during pregnancy. Left: the results of Western blot; Right: histogram of relative protein levels. * *p* < 0.05, compared with control; ** *p* < 0.01, compared with control.

**Figure 7:**
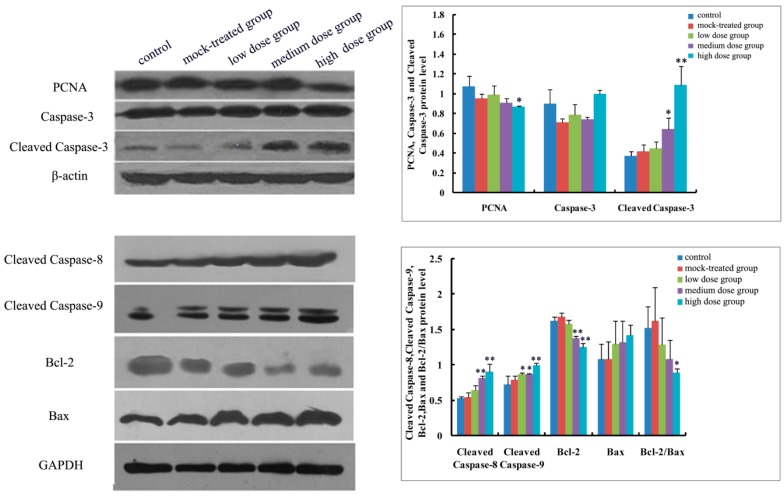
Expressions of apoptosis-related proteins (Caspase-3, Cleaved Caspase-3, Cleaved Caspase-8, Cleaved Caspase-9, Bcl-2, Bax and Bcl-2/Bax) and PCNA in cerebral cortex of mice offspring on postnatal day 14 after maternal exposure to PM_2.5_ during pregnancy. Left: the results of western blot; Right: histogram of relative protein levels. * *p* < 0.05, compared with control; ** *p* < 0.01, compared with control.

**Figure 8:**
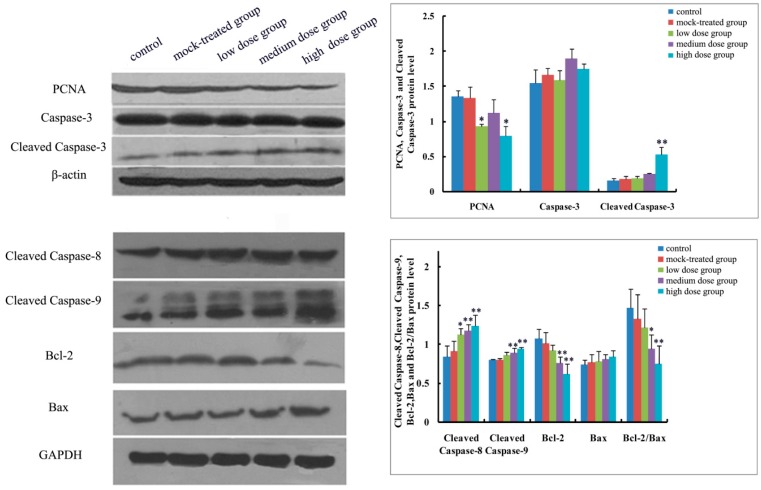
Expressions of apoptosis-related proteins (Caspase-3, Cleaved Caspase-3, Cleaved Caspase-8, Cleaved Caspase-9, Bcl-2, Bax and Bcl-2/Bax) and PCNA in cerebral cortex of mice offspring on postnatal day 21 after maternal exposure to PM_2.5_ during pregnancy. Left: the results of western blot; Right: histogram of relative protein levels. * *p* < 0.05, compared with control; ** *p* < 0.01, compared with control.

**Figure 9:**
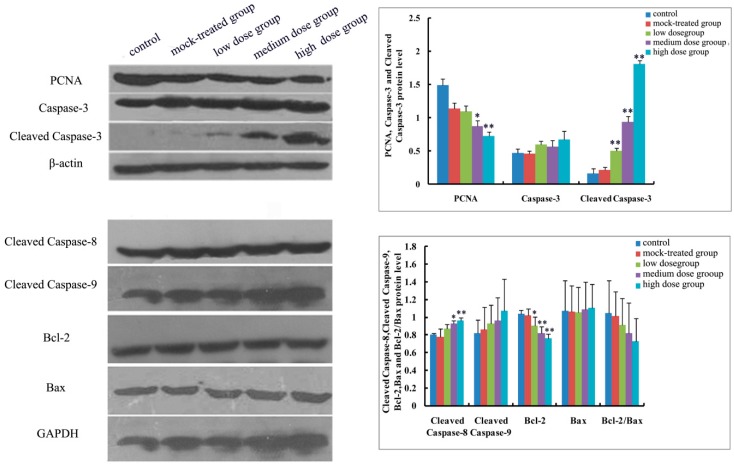
Expressions of apoptosis-related proteins (Caspase-3, Cleaved Caspase-3, Cleaved Caspase-8, Cleaved Caspase-9, Bcl-2, Bax and Bcl-2/Bax) and PCNA in cerebral cortex of mice offspring on postnatal day 30 after maternal exposure to PM_2.5_ during pregnancy. Left: the results of western blot; Right: histogram of relative protein levels. * *p* < 0.05, compared with control; ** *p* < 0.01, compared with control.

As to mRNA level and protein expression of caspase-3, there was no significant difference among the PM_2.5_-treated groups compared with the control (Figure 4A and Figure 5, Figure 6, Figure 7, Figure 8 and Figure 9). In comparison with the control, the protein expression of cleaved caspase-3 increased with exposure dosage at each time point, especially in the high-dosage group. Both mRNA level of caspase-8 and protein expression of cleaved caspase-8 increased with exposure dosage at each time point (Figure 4B and Figure 5, Figure 6, Figure 7, Figure 8 and Figure 9) compared with the control, with significant increase especially in the high-dosage group. mRNA level of caspase-9 increased with exposure dosage at each time point and protein expression of cleaved caspase-9 significantly increased with exposure dosage at each time point except postnatal day 30, in comparison with the control (Figure 4C and Figure 5, Figure 6, Figure 7, Figure 8 and Figure 9).

#### 2.4.2. Effects of Exposure to PM_2.5_ on mRNA Levels and Protein Expressions of Bcl-2 and Bax

mRNA level and protein expression of Bcl-2 decreased at each time point with exposure dosage (Figure 4D and Figure 5, Figure 6, Figure 7, Figure 8 and Figure 9), while there was no significant change of mRNA level or protein expression of Bax at each time point for any dosage group, compared with the control (Figure 4E and Figure 5, Figure 6, Figure 7, Figure 8 and Figure 9). Both mRNA level and protein expression rates of Bcl-2/Bax decreased at each time point with exposure dosage (Figure 4F and Figure 5, Figure 6, Figure 7, Figure 8 and Figure 9), suggesting that exposure to PM_2.5_ during pregnancy might exert effects on neuronal apoptosis in the cerebral cortex of mice offspring via changing the balance between Bcl-2 and Bax.

#### 2.4.3. Effects of Exposure to PM_2.5_ on Protein Expression of PCNA

Protein expression of PCNA decreased with exposure dosage at each time point, statistically significantly different between high-dosage group and control (Figure 5, Figure 6, Figure 7, Figure 8 and Figure 9), which indicated that exposure to PM_2.5_ during pregnancy could suppress the expression of PCNA and then exert effects on proliferation of neurons in the cerebral cortex of mice offspring.

### 2.5. Results of Behavioral Experiments in Mice Offspring 

#### 2.5.1. Results of Open Field Test

**Figure 10:**
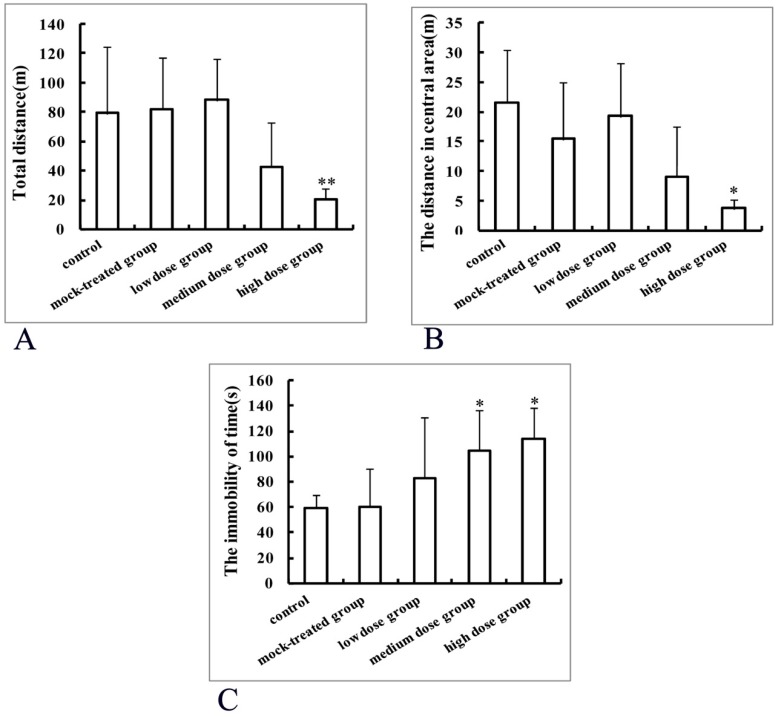
Behavioral effects of maternal exposure to PM_2.5_ during pregnancy on mice offspring in open field test (**A**,**B**) and tail suspension experiment (**C**). * *p* < 0.05, compared with control; ** *p* < 0.01, compared with control.

Mice offspring from the control group traveled a total distance of 79.54 ± 88.80 m within 5 min and the total distance shortened with exposure dosage, with the total distance significantly different from that of high-dosage group (20.24 ± 7.72 m) (*p* < 0.01, Figure 10A). 

Compared with the control group, the activity time in the central area decreased with dosage, but there was no statistically significant difference. Distances traveled in the central area decreased with dosage, with statistically significant difference between the high-dosage and control groups (*p* < 0.05, Figure 10B). Moreover, spontaneous activities reduced with dosage.

#### 2.5.2. Results of Tail Suspension Experiment

The cumulative immobility time durations in tail suspension experiment were 60.33 ± 30.26 s, 59.00 ± 10.18 s, 83.13 ± 47.17 s, 104.45 ± 31.50 s, and 113.89 ± 24.01 s, corresponding to the control, mock-treated, low-dosage, medium-dosage, and high-dosage groups, respectively. The cumulative immobility time durations in the medium and high-dosage groups were significantly prolonged, compared with the control (*p* < 0.05, Figure 10C), with no significant difference among the control, mock-treated, and low-dosage groups.

## 3. Discussion

A PM_2.5_ trachea drip animal model for pregnant mice was established in our preliminary animal experiments where it was found that weight gains and gestation days decreased, along with pathological changes such as inflammation in heart, liver, lung, kidney, and other main organs occurred with exposure dosage of PM_2.5_ [21,22]. Exposure to PM_2.5_ during pregnancy resulted in many adverse pregnancy outcomes such as slow weight gain in pregnant mice, along with reduced number and body weight loss of newborn mice [21]. After entering into maternal blood circulation, PM_2.5_ might interfere with maternal normal metabolism, delay placental growth, reduce the placental nutrient supply and gas exchange, or exert indirect effects on the normal systematic development of fetal mice through a variety of mechanisms including oxidative stress, inflammation, and dysfunction of the placenta resulting in adverse pregnancy outcomes [15,23,24].

After exposure to PM_2.5_, adult mice demonstrated impaired abilities of learning and memory, anxiety- and depression-like behaviors, suggesting that particulate matter pollutants could exert adverse effects on the central nervous system, emotional reactions, and cognitive competence by activating reactive oxygen species (ROS) and proinflammatory cytokine pathways [25]. However, studies concerning the developmental toxicity of exposure to PM_2.5_ during pregnancy to nervous system remain limited [26,27,28]. In particular, very few animal experiments involved the effects and mechanisms of prenatal exposure to PM_2.5_ on the development of the central nervous system in mice offspring [29].

Exposure to PM_2.5_ possessed potential neurotoxicity and could penetrate various biological barriers including blood-gas, placental, and blood-brain barriers in mammals [14], causing inflammations and oxidative stress in the brain [30]. The brain is particularly sensitive to oxidative stress and exhibits morphological changes in the cerebral cortex with the increased exposure dosage of PM_2.5_ after peripheral immune stimulation.

A great deal of oxygen free radicals could result from brain impairment and lead to neuronal base damages which could be repaired by the PCNA-dependent pathway [31]. Protein expression of PCNA (a DNA repair protein) decreased with PM_2.5_ exposure dosage at each time point in mice offspring in the present work. Thus, it could be speculated that the decreased expression of PCNA made its DNA base repair function reduced or lost correspondingly and caused partial genetic damages missing prompt and effective repair, which further aggravated the damages of DNA and resulted in irreversible process and even cell death.

Apoptosis, firstly put forwarded by Kerr et al. [32], plays a key role in the development of the nervous system and homeostasis of adult nervous system. Therefore, abnormal neuronal apoptosis might exert serious effects on the nervous system [33]. In the present study, the rate of TUNEL-positive cells increased significantly with the exposure dosage of PM_2.5_ during pregnancy. The effects of exposure to PM_2.5_ on apoptosis of the cerebral cortex neurons were also confirmed by transmission electron microscope (TEM) examination which exhibited indistinct mitochondrial cristae, myeloid like degeneration, and overflown nuclear chromatin. This might affect the mitochondrial function (membrane potential and permeabilization), inhibition or activation of apoptosis-related molecules, leading to dysregulation of apoptosis via the mitochondrial pathway [34,35]. It could be inferred that abnormal apoptosis of cortical neurons might be related to the induced neurotoxicity. Decreased synaptic gap of cortical neurons, number of presynaptic synaptic vesicles, and density of presynaptic synapses occurred in mice offspring after exposure to PM_2.5_. Synaptic plasticity can affect the brain functions such as development of the nervous system, damage repair, learning, and memory, and thus plays a vital role in the pathogenesis of cognitive impairments [36]. Neuronal damages and alterations in synaptic plasticity are the pathophysiological basis for the development of psychiatric disorders.

Apoptosis involves two pathways: endogenous and exogenous. Endogenous (mitochondrial) pathway can regulate apoptosis initiation genes (Bax or Bcl-2) and caspase-9 to stimulate the release of mitochondrial cytochrome C into cytoplasm followed by combination with apoptosis peptidase activation factor 1(APAF-1) and then induce apoptosis. As to exogenous (transmembrane) pathway, FasL and tumor necrosis factor alpha (TNF-α) act on the corresponding receptors and lead to apoptosis under the stimulation of apoptosis signals [37,38,39]. Bcl-2 protein family is a key regulator of apoptosis and Bax can promote apoptosis while Bcl-2 can inhibit apoptosis, with ratio changes of Bcl-2/Bax directly reflecting the level of apoptosis [40,41]. When stimulated by upstream apoptosis signal, the initiation factors of caspase-8 and caspase-9 are cleaved and activated by other kinases, and the apoptosis signal is then transmitted to the cascade execution factor caspase-3 to induce apoptosis [42]. The results in the present work suggested that effects of exposure to PM_2.5_ during pregnancy on the development of cerebral cortex in mice offspring could be achieved by up-regulating the expressions of Caspase-3, Caspase-8, and Caspase-9, and down-regulating the expression of Bcl-2. Since Bcl-2 could form a heterodimer with Bax, the protein expressions ratio of Bcl-2 to Bax might eventually break the balance and participate in the induction of apoptosis [43]. Furthermore, the effects of exposure to PM_2.5_ during pregnancy on neuronal apoptosis in cerebral cortex of mice offspring would persist. 

An open field test is a classical method to evaluate the motor function and anxiety state of rodent experimental animals [44]. The statistically significantly different results of the open field test between the control and the high-dosage group suggested that long-term exposure to a high dosage of PM_2.5_ during pregnancy might have certain effects on the motor function of mice offspring. The tail suspension test is widely used to evaluate depression-like behavior. In the present work, an immobile posture based on a stress that the tail could not bear was established [45,46] to acquire the immobility time duration and evaluate effects of exposure to PM_2.5_ during pregnancy on depression in mice offspring. The significantly prolonged immobility time duration in the high-dosage group indicated that exposure to PM_2.5_ in early life could induce depression-like behavior in adult mice offspring.

The aforementioned results suggested that exposure to PM_2.5_ during pregnancy could lead to expression changes of apoptosis-related indicators in the cerebral cortex and subsequently then induce apoptosis. Therefore, exposure to PM_2.5_ during pregnancy might break the balance between neurogenesis and apoptosis in the cerebral cortex by inducing neuronal apoptosis, which might be an intrauterine cause of adult depression susceptibility. A relatively low dosage of fine particles could result in certain alterations of those sensitive indicators and become a potential mutagenic factor which might play a significant role in different periods due to some incentives and therefore lead to corresponding lesions. In summary, the effects of exposure to PM_2.5_ on the growth and development of the cerebral cortex in offspring is a multi-mechanism regulation, multi-gene involvement, multi-stage process. To study and understand its effects on the development of the mouse brain in gestational and offspring mice is of great significance. We aim to advocate vigilance and prevention of harm to pregnant women, fetus, and offspring due to pollution of atmospheric particulate matter, and establish early and specific birth defects monitoring indicators. 

## 4. Materials and Methods 

### 4.1. Sampling and Preparation of PM_2.5_

Atmospheric fine particulate matter samplers were used for collecting urban atmospheric PM_2.5_ in a prefecture-level city in Northern China ranging from December 2015 to January 2016. The filter membranes carrying particulate matters were cut into small pieces and processed in deionized water at low temperature by ultrasonic oscillation four times (30 min for each). Then the elution was gathered and freeze-dried in vacuum and the freeze-dried powders were preserved at −20 °C before being dissolved in PBS and used in uniform solution at certain concentration.

### 4.2. Animal Grouping and Model Preparation

Specific pathogen-free (SPF) Kunming mice, 8 to 9-week old, were purchased from Qingdao Laboratory Animal Center and adaptively fed for a week. Female and male mice were crossbred in a proportion of 2:1 and the next day when vaginal plug appeared was considered as day zero of embryonic development (E0). Pregnant mice were randomly divided into five groups (with 6 mice in each group), namely control, mock-treated, low-dosage, medium-dosage and high-dosage groups, respectively. No additional treatment was applied in the control group while 30 µL of PBS was given via intratracheal instillation in the mock-treated group. Meanwhile, 30 µL of PM_2.5_ suspensions, with concentration as 0.2592 µg/µL, 1.56695 µg/µL and 3.456 µg/µL (corresponding to the PM_2.5_ daily dosage upper limit of 75 g/m^3^, PM_2.5_ red haze warning reference value of 500 g/m^3^ and the PM_2.5_ explosion value of 1000 g/m^3^ in different regions in 2015, respectively, based on the Environmental Air Quality Standard issued by the Ministry of Environmental Protection of China), were dripped into the trachea of pregnant mice from low-dosage, medium-dosage and high-dosage group respectively every 3 days from E0 to parturition (totally seven times) according to a modified rapid mice trachea drip method. Groups of pregnant mice took food and water freely during pregnancy. And all of the experiments (including the following analyses) in the present work were conducted in a random and double-blind manner. This study was approved by Institutional Research Ethics Committee of Weifang Medical University (approval code: 2015266; approval date: December 2015).

### 4.3. Nissle Staining

Once obtained from mice sacrificed on 1st, 7th, 14th, 21st and 30th day after birth, brains were immediately fixed in 4% paraformaldehyde for 3 days, followed by being embedded in paraffin. The paraffin-embedded tissues were cut into 5 μm-thick sections which were subject to subsequent Nissl staining. Quantitative analyses were performed according to a method described previously by Miner et al. [47]. The number of neurons in the cerebral cortex of 0.01 mm^2^ was counted (×40 objective lens and ×10 ocular lens). The longest diameter and the shortest diameter of the positive neurons were measured (×40 objective lens and ×10 ocular lens) and the average value was regarded as the cell diameter. Three slices were selected from each group and five field of views were selected from each slice. The data were presented as mean ± standard deviation.

### 4.4. TEM Observation

Cerebral cortex obtained from mice offspring were cut into cubes of about 1 mm^3^ and fixed in 2.5% glutaraldehyde solution. After being stained with both uranyl acetate and lead citrate, the prepared ultrathin sections were washed using deionized water and then observed by transmission electron microscope (TEM) (HT7700, Hitachi, Tokyo, Japan). A quantitative analysis was performed. Ten fields of view for each TEM figure were selected randomly to quantify the number of presynaptic vesicles. The data were presented as mean ± standard deviation.

### 4.5. TUNEL

Mice offspring were perfused transcardially using 4% paraformaldehyde with 0.1 M PBS as solvent. Then brains were isolated and dehydrated with sucrose solutions (20–30%, *w*/*w*), followed by frozen section. Slides, placed in citrate buffer of 0.1 M, were subject to microwave irradiation for 5 min and subsequent rinsing with 0.1 M PBS (pH 6.0) three times. TUNEL reaction mixture, consisting of 10% enzyme solution and 90% label solution (*v*/*v*) (Roche, Shanghai, China), was dropped onto the sections which were then placed in a sealed container at 37 °C for 60 min in the dark. After being rinsed with 0.1 M PBS three times, sections were stained using Hoechst (1:1000) at 37 °C for 30 min in the dark, followed by anti-quenching mounting and fluorescence microscopic observation. Label solution was used as negative control to ensure the specificity of TUNEL. Apoptotic index (AI) was calculated as (TUNEL-positive cell number)/(total cell number) × 100%.

### 4.6. Real-Time Quantitative PCR

Total RNA was extracted from the cerebral cortex tissues of each group at different time points using Trizol reagent (Invitrogen, Carlsbad, CA, USA) and corresponding cDNA was synthesized using a 1st Strand cDNA Synthesis Kit (Takara, Otsu, Japan). The cDNA products were kept at −20 °C prior to use. The primers for target and internal reference (β-actin) genes were designed using a Primer5 software and synthesized by Takara. Then mRNA levels of target and internal reference genes were analyzed in triplicate using a Bio-Rad CFX Manager 3.1 real-time quantitative PCR system(Bio-Rad Laboratories, Hercules, CA, USA), with total reaction volume as 10 μL containing approximately 50 ng cDNA, 5 μL SYBR premix, 10 μM primer each and diethylpyrocarbonate (DEPC) processed deionized water. Positive and negative controls were also used for the accuracy of experimental results. Relative mRNA levels of target genes were calculated using the 2^−△△*C*t^ method. The primers used in the present work were shown in Table 2.

### 4.7. Western Blot

Samples of cerebral cortex were separated from newborn mice on postnatal days 1, 7, 14, 21, and 30, respectively, and homogenized at 4 °C in lysis buffer consisting of 50 mM NaCl, 1 mM ethylene diamine tetraacetic acid (EDTA) (pH 8.0), 50 mM Tris-HCl (pH 7.4), 1% Triton-100 and 100 µg/mL PMSF. Lysates were centrifuged at 12,000× *g* for 15 min to obtain the supernatant and protein concentrations were determined by BCA method. Protein samples were separated by 10% SDS-PAGE electrophoresis and then transferred onto PVDF membrane (Millipore, Billerica, MA, USA) at a voltage of 90 V for 1 h. The transferred proteins were blocked using 5% skim milk in TBST buffer on a shaker for 2 h at ambient temperature, followed by incubations with TBST diluted mice PCNA (1:500, Abcam, Boston, MA, USA) primary monoclonal antibody or caspase-3 rabbit primary polyclonal antibody (1:100, Abcam), or cleaved caspase-3/cleaved caspase-9 rabbit primary polyclonal antibody (1:500, CST, Beverly, MA, USA), or cleaved caspase-8 rabbit primary monoclonal antibody (1:300, Abcam), or rabbit Bcl-2 (1:500, CST) primary monoclonal antibody or rabbit Bax primary polyclonal antibody (1:300, CST), respectively at 4 °C overnight. Then the proteins were incubated with TBST diluted goat-anti-mice IgG (1:2000, CST) or goat-anti-rabbit IgG (1:2000, CST) on a shaker for 2 h at ambient temperature accordingly, followed by the analyses performed using a chemiluminescence detection kit (thermo, Rockford, IL, USA) in darkroom. GAPDH and β-actin (for PCNA, caspase-3 and cleaved caspase-3) were used as internal references (1: 3000, MULTI Sciences, Shanghai, China). The experiment was replicated three times and gray density analyses were performed using a gel image analyzer (UVP, Upland, CA, USA).

### 4.8. Behavioral Experiments

Mice offspring were acclimatized to the experimental conditions overnight and behavioral experiments were conducted between 9 a.m. and 4 p.m. using mice offspring that were 6 weeks old. Residual odors were removed by cleaning the behavioral equipment with 75% ethanol. Open field and the tail suspension tests were conducted using a Locomotion Activity Video Analysis System (Jiliang Software, JLBehv-LAM-1, Shanghai, China).

#### 4.8.1. Open Field Test

Each mouse was placed at the midpoint of a Plexiglas cage (30 cm × 30 cm × 30 cm) equipped with room light for 5 min. Parameters, including total travelled distance, time spent in the central area (20 cm × 20 cm) and activity path were recorded.

#### 4.8.2. Tail Suspension Experiment

A small clip, connected to the top of tail suspension test operation box, was used to fasten the adhesive tape wrapped around the tail end of mouse which was overhung for totally 6 min, with a distance of 4~5 cm between mouse head and box bottom. Time duration for mice tail suspension was recorded during the last 4 min. Tail suspension refers to completely immobile limbs or merely slight feet movements.

### 4.9. Statistical Analysis

All of the data were reported as mean values ± standard deviation (SD). Multiple comparisons were analyzed by one-way ANOVA using a SPSS19.0 software (SPSS Inc., Chicago, IL, USA). The comparisons were conducted by the least significant difference test (LSD-t) for homogeneous variance or Games-Howell test for inhomogeneous variance. *p* < 0.05 was considered to be statistically significant. 

## 5. Conclusions

In conclusion, our results showed that maternal exposure to PM_2.5_ during pregnancy induced mitochondrial dysfunction and neuronal apoptosis in the cerebral cortex of mice offspring. The exposure also exerted certain negative effects on the later neurobehaviors of mice offspring. Therefore, exposure to PM_2.5_ during pregnancy should receive people’s high attention. They are advised to pay close attention to the air quality, seek protection when needed, and avoid long-term exposure to PM_2.5_ especially at high dose. These actions may help avoiding the adverse development of fetus. 

## Figures and Tables

**Table 1:** Parameters of neurons in cerebral cortex.

Group	Diameter of Neurons (μm)	Number of Neurons
Mock-treated group	18.25 ± 1.09	59 ± 4.58
Low-dosage group	17.66 ± 0.88	58 ± 5.29
Medium-dosage group	14.58 ± 1.02 **	45.33 ± 6.43 *
High-dosage group	14.34 ± 1.14 **	38 ± 7.21 **

* compared with mock-treated group *p* < 0.05, ** compared with mock-treated group *p* < 0.01.

**Table 2:** Primers used for real-time quantitative PCR.

Target	Primer Sequence (5′–3′)
Caspase-3	F: CTGGACTGCGGTATTGAGAC
	R: CCGGGTGCGGTAGAGTAAGC
Caspase-8	F: TGCTTGGACTACATCCCACAC
	R: TGCAGTCTAGGAAGTTGACCA
Caspase-9	F: TCCTGGTACATCGAGACCTTG
	R: AAGTCCCTTTCGCAGAAACAG
Bcl-2	F: GTCGCTACCGTCGTGACTTC
	R: CAGACATGCACCTACCCAGC
Bax	F: TGAAGACAGGGGCCTTTTTG
	R: AATTCGCCGGAGACACTCG
β-actin	F: GGCTGTATTCCCCTCCATCG
	R: CCAGTTGGTAACAATGCCATGT

## Abbreviations

EPA	American Environmental Protection Administration
TUNEL	Terminal Deoxynucleotidyl transferase dUTP Nick end Labeling
PBS	Phosphate Buffered Solution
TEM	Transmission Electron Microscope
